# Genotype Distribution and High-Risk Factors Analysis of Group B Streptococcus in Late-Stage Pregnant Women in the Linyi Region

**DOI:** 10.1155/ijm/9910073

**Published:** 2024-12-19

**Authors:** Weiping Zhou, Xiaoyan Chen, Jie Chen, Xiuhua Zheng, Xueqiang Zhang, Yubin Chen, Yuehua Pan, Chunling Ma

**Affiliations:** ^1^Clinical Laboratory, Women's & Children's Health Care Hospital of Linyi, Linyi 276000, Shandong, China; ^2^Key Laboratory of Birth Defects, Women's & Children's Health Care Hospital of Linyi, Linyi 276000, Shandong, China

**Keywords:** genotypic distribution, Group B Streptococcus, late pregnancy, risk factors, virulence genes

## Abstract

**Objective:** To understand the colonization status of Group B Streptococcus (GBS) in the reproductive tract of pregnant women in the Linyi region, the drug resistance, genotype distribution, and molecular epidemiological characteristics of GBS, and to explore the high-risk factors for GBS infection in late-stage pregnant women.

**Methods:** A total of 3269 pregnant women at 35–37 weeks of gestation who visited the Obstetrics Department of Linyi Maternal and Child Health Hospital from January 2019 to December 2021 were selected as the study subjects. Vaginal and rectal swabs were collected for GBS culture. Based on the culture results, they were divided into positive and negative groups. The high-risk factors such as age, BMI index, education level, pregnancy vomiting, and liver function indicators of the two groups were analyzed. Drug sensitivity test, multilocus sequence typing (MLST) gene typing, and virulence factor detection were performed on GBS (+) strains.

**Results:** The infection rate of GBS in the reproductive tract of pregnant women in late pregnancy in the Linyi region was 7.07% (231/3269). The analysis of high-risk factors showed that having a college degree or above and absence of pregnancy vomiting; elevated levels of alanine aminotransferase, albumin, globulin, direct bilirubin, glutamyl transferase, and total bile acids; and decreased levels of alkaline phosphatase and lactate dehydrogenase were high-risk factors for GBS infection (*p* < 0.05). The MLST results showed that a total of 189 GBS strains were identified with 20 genotypes, the top four being ST10 type (25.40%), ST19 type (17.99%), ST529 type (13.76%), and ST862 type (12.70%). The 20 ST came from 8 CCs, with the main CC groups being CC12 (29.11%), CC19 (24.87%), CC103 (18.00%), and CC327 (13.76%). GBS strains showed high sensitivity to vancomycin, penicillin, and levofloxacin, all being 100%; sensitivity to erythromycin, clindamycin, compound novobiocin, and tetracycline was relatively low; there were statistically significant differences in resistance to erythromycin, clindamycin, and levofloxacin among different genotypes of GBS (*p* < 0.05). The detection rates of GBS virulence factors hylB (81.46%) and scpB (80.98%) were the highest. In ST10 type, > 90% of strains carried bac, bca, hylB, and scpB; in ST19 and ST529, > 90% of strains carried hylB and scpB; and in ST862, > 90% of strains carried CPSIII.

**Conclusion:** The colonization rate of GBS in the reproductive tract of pregnant women in late pregnancy in the Linyi region is 7.07%. Having a college degree or above, absence of pregnancy vomiting, elevated levels of albumin, globulin, direct bilirubin, glutamyl transferase, and total bile acids, and decreased levels of alkaline phosphatase and lactate dehydrogenase are high-risk factors for GBS infection; ST10, ST19, ST529, and ST862 are the main genotypes prevalent in this region; there are regional differences in the distribution of GBS genotypes and CC groups; there are statistically significant differences in the distribution of virulence factors among GBS strains with different MLST genotypes (*p* < 0.05); GBS shows high sensitivity to penicillin drugs and can still be used as the preferred medication for the prevention and treatment of GBS infection.

## 1. Background

Group B Streptococcus (GBS) is widely found in the natural environment and is the most important pathogen-causing mastitis in dairy cows [1]. Human GBS is a common type of cocci bacteria found in the body, frequently colonizing areas such as the female vagina and rectum. While GBS colonization is typically harmless, if a GBS infection is detected during a prenatal examination and shows a strong positive result, it may be associated with specific clinical symptoms. For instance, if GBS infects the uterus, it can lead to premature rupture of the fetal membrane and intrauterine infection of the fetus. During delivery, GBS can also cause neonatal infections, such as pneumonia, septicemia, and meningitis [2]. In adults, GBS infection is characterized by bacteremia, soft tissue infection, and pneumonia. As a result, GBS is the leading cause of neonatal infections and severe infections in pregnant women in many countries [1].

Recent studies show that GB streptococcal infection is on the rise in women of childbearing age worldwide. The rate of infection varies in different parts of the world. For example, the infection rate among pregnant women in the United Kingdom is 14%, and the rate in Washington, D.C., of the United States is 10.8% [3]. African women are at a higher risk of contracting GBS compared to Europeans. However, in some low-income countries in Africa, pregnant women lack access to comprehensive data because they cannot afford diagnostic tests. The cumulative GBS infection rate among women from three communities in Zimbabwe was 60.3% [4]. While Asian women have a slightly lower risk. In mainland China, the colonization rate among pregnant women ranges from 3.7% to 14.52% [5]. Studies have found that the pathogenicity of GBS is closely related to the existence of multiple virulence factors [6]. At present, the virulence factors that have been reported include genes of GBS capsular polysaccharide antigen (CPA) [7, 8], C protein α/α antigen (*bca*), C protein β/β antigen (*bac*), surface protein rib (*Rib*), C5a peptidase (*scpB*), and hyaluronic acid lyase (*hly*) [9, 10].

The aim of this study is to comprehensively investigate the colonization patterns of GBS in the reproductive tract of pregnant women in the Linyi region of China. By identifying high-risk factors for infection during late pregnancy, we seek to provide critical insights into the genotype distribution and drug resistance of GBS within the local population. This study will contribute to better targeted prevention and treatment strategies for maternal and neonatal health in the region.

## 2. Materials and Methods

### 2.1. Materials

A total of 3269 pregnant women who visited the Women's and Children's Healthcare Hospital of Linyi from January 2019 to December 2021 were selected as the study subjects. Vaginal and rectal swabs were collected as the specimens upon admission. Based on the GBS test results, the subjects were divided into the GBS positive group (231 cases) and the GBS negative group (3038 cases). Informed consent was obtained from pregnant women, and the study was approved by the ethics committee of Women's and Children's Healthcare Hospital of Linyi (No. KYL-YXLL-2021013).

The inclusion criteria include the following:

1. Pregnant women at 35–37 weeks of gestation who underwent GBS testing
2. No prior use of antimicrobial drugs
3. No vaginal infections

The exclusion criteria include the following:

1. Incomplete clinical data
2. Recent sexual activity or use of antimicrobial drugs within the past 2 weeks before screening
3. Use of vaginal washes or suppositories within 24 h prior to sampling
4. Coexistence of other malignant diseases or impaired liver or kidney function

### 2.2. Methods

#### 2.2.1. Main Instruments and Reagents

Main instruments were as follows: PCR gene amplifier (Bio-rad DNA Engine Dyad), gel imaging system (Bio-Rad GEL Doc2000), BD Phoenix M50 fully automated bacterial identification instrument (United States of America), and Shanghai Lishen CO_2_ incubator. Main reagents were as follows: bacterial genomic DNA extraction kit (Tiangen Biotech Co., Ltd.), 2 × Taq PCR Mix (Tiangen Biotech Co., Ltd.). PCR primers were synthesized by Shanghai Sangon Biotechnology Services Co., Ltd. PCR products were sequenced by Beijing Liuhe Huada Genomics Institute Co., Ltd. Finished blood agar medium, GBS chromogenic medium, and blood Mueller–Hinton agar were purchased from Zhengzhou Antu Bioengineering Co., Ltd.

#### 2.2.2. Sampling Method

Sterile cotton swabs were used to collect vaginal secretions from the vaginal orifice of pregnant women. After cleaning, another sterile cotton swab was inserted into the vagina about one-third of the way and rotated to collect vaginal secretions. A third cotton swab was placed on the anal sphincter about 2-3 cm from the anus to collect rectal secretions. The sampling procedure was performed gently, and the cotton swabs were placed in sterile tubes and sent to the laboratory for testing within 60 min.

#### 2.2.3. GBS Culture and Identification

The specimens were inoculated on blood agar plates and GBS chromogenic plates using the quadrant streak method. The inoculated plates were placed in a 5% CO_2_ incubator and incubated at 37°C for 18–24 h. If red colonies appeared on the GBS chromogenic plates or if a β-hemolytic zone appeared at the edge of the colonies on the blood agar plates, they were suspected to be GBS. Confirmation of GBS identification was done using the BD Phoenix M50 fully automated bacterial identification instrument.

#### 2.2.4. GBS Drug Susceptibility Testing

The Kirby–Bauer method was used to test the drug susceptibility of penicillin, erythromycin, clindamycin, vancomycin, tetracycline, the combination of fenoterol and novobiocin, and levofloxacin. The clindamycin induction test was performed as follows: 15 μg erythromycin and 2 μg clindamycin disks were placed on blood Mueller–Hinton agar, with the distance between the edges of the two disks ranging from 15 to 26 mm. Inducible resistance to clindamycin was determined when a “D-zone” appeared at the edge of the clindamycin disk adjacent to the erythromycin disk. The interpretation of drug sensitivity was based on the latest edition of the Clinical and Laboratory Standards Institute (CLSI) M100 2021 version.

#### 2.2.5. Multilocus Sequence Typing (MLST) Typing

According to Park JS et al. [11], bacterial genomic DNA was extracted using a genomic DNA extraction kit as a template. PCR was used to amplify 7 housekeeping genes (*adhP, pheS, atr, glnA, sdhA, glcK, and tkt*). The PCR products were sent to Beijing Liuhe Huada Genomics Institute Co., Ltd., for sequencing, and the obtained sequences were aligned on the pubMLST website (https://www.mlst.net/) to determine the strain typing. The concatenated sequences of the housekeeping genes were aligned and compared using MEGA 11.0 software, and the genetic evolutionary relationship was analyzed using the UPGMA method.

#### 2.2.6. Virulence Factor Detection

According to the literature [7], primers were synthesized to detect the virulence genes of GBS, including alpha-C protein gene (*bca*), β-C protein gene (*bac*), and 5C peptide gene (*scpB*, *hylB*, *rib*, and *cpsIII*). The amplified products were detected by 1.0% agarose gel electrophoresis and sent for sequencing.

#### 2.2.7. Statistical Analysis

Data analysis was performed using SPSS 23.0 statistical software. Normally distributed continuous data were expressed as mean ± SD, and the *t*-test was used for comparison between the two groups. Count data were presented as numbers and (%), and the *χ*^2^ test was used. A *p* value < 0.05 was considered statistically significant.

## 3. Results

### 3.1. GBS Infection in Late-Stage Pregnant Women at Our Hospital From 2016 to 2021

**Figure 1:**
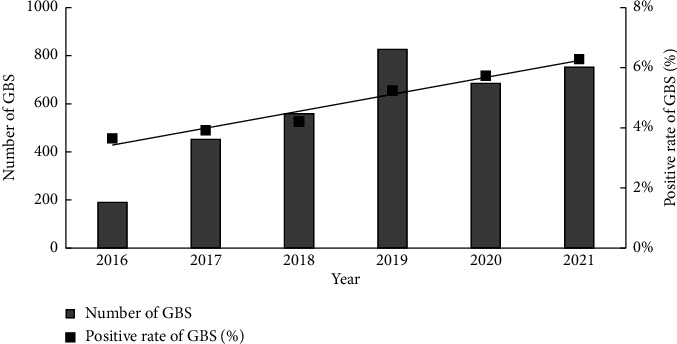
Group B Streptococcus detection rate in late-stage pregnant women at our hospital from 2016 to 2021.

The total number of GBS tests conducted on late-stage pregnant women at our hospital from 2016 to 2021 was 81,349, with 4290 positive cases. The average annual positivity rate was 5.27%. The positivity rate of GBS in late-stage pregnant women showed a gradual increase from 2016 to 2021, and the increase in the positivity rate over these years was statistically significant (*R*^2^ = 0.987, *p*=0.001). The detection rate of GBS infection in late-stage pregnant women at our hospital from 2016 to 2021 is shown in Figure 1.

Based on the total number of GBS screening and the number of positive screening during the third trimester of pregnancy from 2016 to 2021, the positive rate of GBS screening during the third trimester of pregnancy from 2016 to 2021 in the Linyi region was calculated.

### 3.2. GBS Infection and High-Risk Factors in 3269 Cases of Late-Stage Pregnant Women

From January 2019 to December 2021, a total of 3269 specimens were collected, among which 231 were positive for GBS, resulting in a positivity rate of 7.07%. Analysis of the basic characteristics of pregnant women showed that having an education level of college or above and no morning sickness were significant high-risk factors for GBS infection (both *p* < 0.05), as shown in Table 1. Analysis of laboratory indicators as high-risk factors for GBS infection in pregnant women showed that elevated levels of aspartate aminotransferase, albumin, globulin, direct bilirubin, glutamyl transferase, and total bile acids, as well as decreased levels of alkaline phosphatase and lactate dehydrogenase, were significant factors (all *p* < 0.05), as shown in Table 2.

### 3.3. Distribution of GBS MLST Genotypes

**Figure 2:**
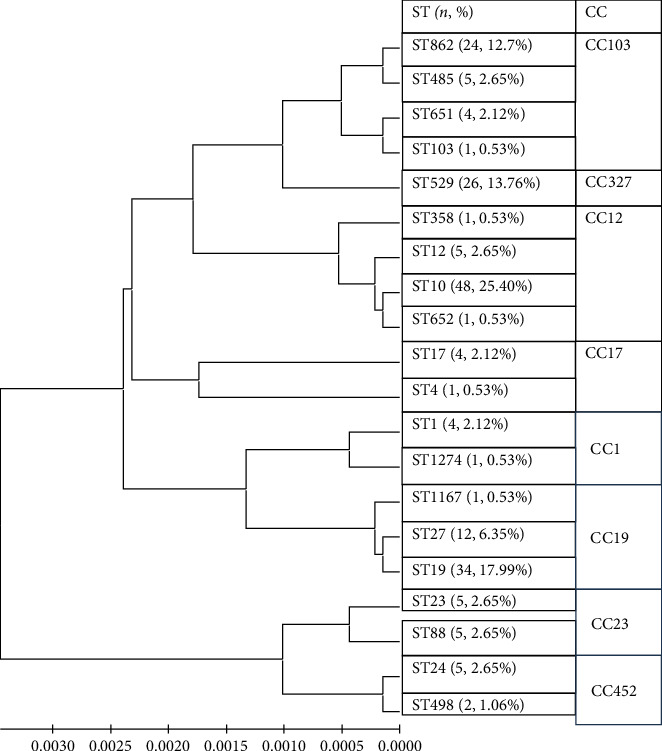
Clustering analysis of 189 Group B Streptococcus strains. ST, sequential type; CC, clone group; *n* represents the frequency and percentage of ST separation. CC uses eBURST version 3.1 software. All ST were analyzed and classified into different clone groups. MEGA7 software was used for clustering analysis with the UPGMA method.

MLST genotyping was performed on successfully recovered 189 *GBS* strains, identifying a total of 20 different ST, the main types were ST10, ST19, ST529, and ST862, accounting for 25.40%, 17.99%, 13.76%, and 12.70%, respectively, as shown in Figure 2. These 20 ST originated from 8 colonial complexes (CCs), with the main CC groups being CC12 (29.11%), CC19 (24.87%), CC103 (18.00%), and CC327 (13.76%).

### 3.4. Sensitivity of GBS to Clinical Drugs

The drug sensitivity test results of 189 GBS strains showed that all detected GBS strains were 100% sensitive to penicillin, vancomycin, ceftriaxone, and cefepime. Sensitivity to erythromycin and clindamycin was relatively low. There were statistically significant differences in resistance to erythromycin, clindamycin, and levofloxacin among different genotypes of GBS, as well as differences in inducible clindamycin resistance (*p* < 0.05). See Table 3.

### 3.5. Distribution of GBS Virulence Factors

**Figure 3:**
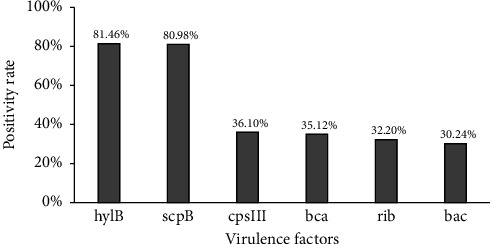
Positivity rates of virulence factors in clinical Group B Streptococcus isolates. A total of 6 virulence factors were detected in 189 strains of Group B Streptococcus isolates. In this study, the 6 bar charts show the percentage of each virulence factor in 189 strains of Group B Streptococcus isolates.

Among the 189 strains of GBS, the detection rate of virulence factors was highest for *hylB* and *scpB*, with positivity rates of 81.46% (167 strains) and 80.98% (166 strains), respectively. The positivity rate for *cpsIII* was 36.10% (74 strains), for *bca* was 35.12% (72 strains), for *rib* was 32.20% (66 strains), and for *bac* was 30.24% (62 strains). See Figure 3.

### 3.6. Distribution of Virulence Factors Among Major ST of GBS

Over 90% of GBS ST10 strains carried *bac, bca, hylB*, and *scpB*; over 90% of ST19 and ST529 strains carried *hylB* and *scpB*; and over 90% of ST862 strains carried *cpsIII*. There was a statistically significant difference in the distribution of virulence factors among different GBS genotypes (*p* < 0.05). See Table 4.

## 4. Discussion

GBS is a commensal bacterium commonly found in the gastrointestinal and vaginal flora of healthy individuals. However, when present in pregnant women, GBS can significantly increase the risk of preterm birth, premature rupture of membranes, and severe neonatal infections due to vertical transmission during delivery [12–14]. This study's finding of a 7.07% colonization rate among late-stage pregnant women in the Linyi region aligns with the previous research but highlights specific regional variations and factors influencing infection risk. The observed infection rate is consistent with rates reported in various provinces in China but lower than those in some northern European countries [5, 15–17]. Notably, the increased detection rate in recent years since the relaxation of China's two-child policy suggests that demographic changes and increased population size may influence infection rates. The evolving population dynamics could contribute to the observed trends in GBS colonization.

Our study identified several high-risk factors for GBS infection, including higher education levels, absence of morning sickness, and specific liver function indicators. These findings diverge from previous studies, such as those by Alfouzan W et al. [18] which reported an inverse relationship between educational level and GBS infection. This discrepancy could be due to different study populations, methodologies, or confounding variables not accounted for in the previous research.

### 4.1. Confounding Factors

1. Socioeconomic status and healthcare access: The association between higher education levels and increased GBS risk might be confounded by the socioeconomic status. Higher education is often linked with better healthcare access, potentially leading to more frequent testing and higher detection rates. Socioeconomic factors might also influence lifestyle and health behaviors that affect GBS colonization.
2. Health conditions and nutritional status: The absence of morning sickness could be indicative of underlying health conditions or differences in immune responses that affect GBS susceptibility. In addition, variations in the nutritional status among women with higher education levels might influence both liver function indicators and infection risk.
3. Pre-existing conditions and medication use: Elevated or decreased liver function indicators might reflect pre-existing health conditions that predispose individuals to GBS infection. Medications affecting liver function could also confound the observed associations, making it crucial to control for these factors in future analyses.

The study's genotypic analysis revealed a high genetic diversity of GBS strains in the Linyi region, with ST10, ST19, ST529, and ST862 being most prevalent. This finding aligns with regional differences observed in other studies, such as those by Guan et al. [19], Kang et al. [20], Gherardi et al. [21], and Teatero et al. [22]. The identification of different CCs and significant regional variations underscores the importance of localized epidemiological studies.

The high sensitivity of GBS strains to penicillin and vancomycin, coupled with resistance to erythromycin and clindamycin, emphasizes the need for careful antibiotic selection, particularly for penicillin-allergic patients. The significant differences in antibiotic resistance among different genotypes further highlight the necessity for routine susceptibility testing and targeted treatment strategies [23].

The study's analysis of virulence factors revealed a distribution pattern that correlates with specific GBS genotypes, similar to the findings reported by Liang et al. [24]. The detection of virulence factors such as *hylB* and *scpB* in high frequencies among various genotypes reinforces the role of these factors in GBS pathogenicity.

### 4.2. Limitations and Future Directions

The study's limitations include the lack of multivariable regression analysis to fully account for potential confounders influencing GBS colonization. The increasing GBS carriage rate in the local area may be influenced by unstudied population-specific factors, necessitating further research to elucidate the reasons behind this trend.

## 5. Conclusion

This study contributes to our understanding of GBS epidemiology in the Linyi region by identifying key risk factors, genotypic diversity, and antibiotic resistance patterns. The findings underscore the importance of considering confounding variables in the future research and highlight the need for localized prevention and treatment strategies. Continued surveillance and tailored interventions will be essential for effectively managing GBS infections in the perinatal period and improving maternal and neonatal health outcomes.

## Figures and Tables

**Table 1:** Analysis of basic characteristics as influencing factors for Group B Streptococcus colonization in pregnant women.

Group	Positive (*N* = 231)	Negative (*N* = 3038)	*χ* ^2^ value	*p* value
*Age (year)*				
< 35	224 (96.97%)	2879 (94.77%)	2.162	0.141
≥ 35	7 (3.03%)	159 (5.23%)		

*BMI*				
< 25	186 (80.52%)	2458 (80.91%)	0.021	0.885
≥ 25	45 (19.48%)	580 (19.09%)		

*Education level*				
Below college degree	86 (37.23%)	1853 (60.99%)	**50.240**	**0.001**
College degree and above	145 (62.77%)	1185 (39.01%)		

*Morning sickness*				
Present	103 (44.59%)	1620 (53.32%)	**6.572**	**0.010**
Absent	128 (55.41%)	1418 (46.68%)		

*Note:* Data presented as the number of cases (percentage); positive group versus negative group, analyzed by chi-square test. Bold values indicate that there is a significance of the analyte between positive group and negative group.

Abbreviation: BMI = body mass index.

**Table 2:** Analysis of laboratory indicators as high-risk factors for Group B Streptococcus colonization in pregnant women.

Laboratory indicator	Experimental group (*n* = 231)	Control group (*n* = 3038)	*t* value	*p* value
AST (U/L)	11.90 ± 6.46	13.90 ± 11.25	**2.668**	**0.008**
ALT (U/L)	16.06 ± 4.41	16.37 ± 7.07	0.657	0.511
TP (g/L)	61.36 ± 14.60	60.98 ± 19.86	0.285	0.776
ALB (g/L)	38.00 ± 7.87	41.73 ± 4.79	**10.782**	**0.001**
GLOB (g/L)	28.64 ± 3.83	29.49 ± 4.54	**2.771**	**0.006**
TBIL (μmol/L)	7.92 ± 3.32	8.35 ± 8.35	0.778	0.437
DBIL (μmol/L)	1.80 ± 2.32	2.34 ± 2.71	**2.947**	**0.003**
IBIL (μmol/L)	6.65 ± 2.76	6.98 ± 3.06	1.591	0.112
ALP (U/L)	98.04 ± 58.26	67.02 ± 49.63	**9.038**	**0.001**
GGT (U/L)	13.57 ± 12.71	16.39 ± 15.67	**2.669**	**0.008**
CHE (U/L)	5726.70 ± 1591.96	5872.24 ± 2199.37	0.986	0.324
TBA (μmol/L)	333.32 ± 1591.16	635.69 ± 1981.84	**2.264**	**0.024**
LDH (U/L)	149.76 ± 40.28	138.13 ± 50.06	**3.447**	**0.001**

*Note:* Data presented as mean ± SD; Bold values indicate that there is a significance of the analyte between the positive group and negative group.

Abbreviations: ALB = albumin; ALP = alkaline phosphatase; ALT = alanine aminotransferase; AST = aspartate aminotransferase; CHE = cholinesterase; DBIL = direct bilirubin; GGT = glutamyl transferase; GLOB = globulin; IBIL = indirect bilirubin; LDH = lactate dehydrogenase; TBA = total bile acids; TBIL = total bilirubin; TP = total protein.

**Table 3:** Drug resistance of Group B Streptococcus to clinical drugs in different genotypes (ST).

Antibiotics	Overall drug resistance rate (*n* = 189)	ST10 (*n* = 48)	ST19 (*n* = 34)	ST529 (*n* = 26)	ST862 (*n* = 24)	*χ* ^2^ value	*p* value
E	150 (71.17%)	46 (95.83%)	23 (67.65%)	25 (96.15%)	12 (50.00%)	28.826	0.001
DA	158 (77.07%)	46 (95.83%)	26 (76.47%)	24 (92.31%)	16 (66.67%)	13.658	0.003
LEV	91 (44.39%)	47 (97.92%)	34 (100.00%)	0	0	127.87	0.001
D (+)	30	1 (3.33%)	6 (20.00%)	13 (43.33%)	0	35.387	0.001

*Note:* DA, clindamycin; D (+), clindamycin induction test is positive; *n* represents the frequency of ST separation.

Abbreviations: E, erythromycin; LEV, levofloxacin; ST, sequence type.

**Table 4:** Distribution of virulence factors in different serotypes (ST) of Group B Streptococcus.

Virulence factors	ST10 (25.20%)	ST19 (17.99%)	ST529 (13.76%)	ST862 (12.70%)	*χ* ^2^ value	*p* value
bac	100% (48/48)	2.94% (1/34)	0	4.17% (1/24)	135.955	0.001
bca	97.92% (47/48)	0	3.85% (1/26)	0	135.942	0.001
rib	0	55.88% (19/34)	84.61% (22/26)	4.17% (1/24)	87.431	0.001
hylB	91.67% (44/48)	94.12% (32/34)	96.15% (25/26)	8.33% (2/24)	76.024	0.001
scpB	95.83% (46/48)	94.12% (32/34)	100% (26/26)	4.17% (1/24)	109.326	0.001
cpsIII	0	73.53% (25/34)	15.38% (4/26)	95.83% (23/24)	97.519	0.001

*Note:* The major types of carrying virulence factors were analyzed. The data outside parentheses represented the detection rate of virulence factors in this genotype, and the data in parentheses represented the ratio of positives to the total number. *p* < 0.05 was considered statistically significant.

## Data Availability

The data that support the findings of this study are available from the corresponding author upon reasonable request.
